# How Radical Is Embodied Creativity? Implications of 4E Approaches for Creativity Research and Teaching

**DOI:** 10.3389/fpsyg.2019.02372

**Published:** 2019-10-22

**Authors:** Laura H. Malinin

**Affiliations:** Department of Design and Merchandising, College of Health and Human Sciences, Colorado State University, Fort Collins, CO, United States

**Keywords:** embodied cognition, embodied creativity, embodied design, 4E creativity, enactive cognition, distributed cognition, social creativity, embodied metaphors

## Abstract

Modern ideas of embodiment have been influential in cognitive science for the past several decades, yet there is minimal evidence of embodied cognition approaches in creativity research or pedagogical practices for teaching creativity skills. With creativity research in crisis due to conceptual, methodological, and theoretical issues, radical embodied cognitive science (RECS) may offer a framework to move the field forward. This conceptual analysis examines the current state of creativity research from the 4E (embodied, embedded, enactive, and extended) cognition and RECS perspectives. Two streams are critiqued for their potential to further knowledge about the development of creative expertise and inform educational practices. Promising directions for future research is discussed, including ways dynamical systems approaches, such as those used in improvisational and musical creativity, might yield new insights about how people develop creative expertise and help address the “higher order thinking” criticisms of RECS.

## Introduction

Modern ideas of embodiment can be traced back several decades (Brooks, 1991; Varela et al., 1991; Lakoff and Johnson, 1999), and the notion that cognition may be “radically” embodied has been at the forefront of cognitive science debate since the early 21^st^ century (Thompson and Varela, 2001; Chemero, 2009). Radical embodied cognitive science (RECS) proposes that *cognition is for action*, and thus is best understood in terms of person-environment dynamics, an alternative to computational cognition (CC) models describing rule-based information processing and manipulation of abstract mental representations (Chemero, 2009, pp. 28–44). Importantly, the RECS perspective suggests that how we learn and develop expertise is shaped, constrained, and enacted through exploration and interaction with our physical environment. Despite the potential for RECS to yield new insights into how people acquire creative abilities, the field of creativity research has not given much attention to the role of the body or its physical context (Glăveanu, 2014b; Malinin, 2016). Given that creative process research has made little progress in recent years (Fryer, 2012; Glăveanu, 2014a) and continues to suffer from lack of relevance for informing educational practices or learning environments (Plucker et al., 2004; Barbot et al., 2015), researchers may benefit from adopting an action-oriented and physically-situated perspective. This paper examines the current state of creativity research with respect to embodiment theories in order to focus scholarly discussion around the potential for an embodied perspective to help address conceptual, methodological, and theoretical barriers limiting practical application in educational settings.

As a research area within cognitive science, embodied cognition (EC) is an interdisciplinary effort with diverse and sometimes conflicting perspectives (Gallagher, 2015a). Generally speaking, there are key commonalities among the many existing theories. Foremost is that the body plays a role in shaping perception and cognition. To what extent remains debated. Theories grounded in philosophical perspectives (e.g., Husserl, Merleau-Ponty, and Heidegger) typically focus on ways the body constrains cognition (Borghi and Cimatti, 2010) whereas RECS, with concepts traced back to Gibson’s (1979) ecological theory of perception, is concerned with how cognition is enacted through, or partially constituted by, bodily and environmental processes (Chemero, 2009, pp. 17–44). The second commonality is rejection of the computational “classical sandwich” (Hurley, 2002, p. 401) model of the mind, which describes cognition as staged information processing: perception, then cognition, and finally action. The embodied approach theorizes a complementary relationship between action and perception, and is concerned with how action influences perception and cognition. RECS takes this idea further, arguing that cognition is best understood as a dynamical system encompassing brain, body, and world (Thompson and Varela, 2001, p. 418). Central to RECS is that cognition is not wholly in-the-head, but also involves the body and aspects of the environment. The core conceptual issue between EC and RECS theories concerns whether they complement or oppose the classical computational theory of mind (Gallagher, 2015a). Thusly, a major criticism of RECS is that it may not be sufficient to describe higher-order thinking without relying to some degree on mental representation (Chemero, 2009, p. 43).

Higher-order thinking is a concept based on learning taxonomies, which are used by educators to describe learning behaviors and distinguish levels of cognition. The well-known Bloom’s Taxonomy was revised in 2001 to describe creativity as the most complex (highest-order) process in the knowledge hierarchy (Anderson et al., 2001). Not long after, the Partnership for 21^st^ Century Learning identified four learning skills and competencies essential for a knowledge economy: creativity, collaboration, communication, and critical thinking (4Cs). Researchers generally agree that creativity involves a unique combination of ordinary cognitive processes, such as divergent and convergent thinking, conceptual combination (analogical and metaphorical thinking), mental imagery, and analogical reasoning (Ward and Saunders, 2003; Runco, 2007b). It is also understood to be a teachable skill (Scott et al., 2004; Ward and Kolomyts, 2010), however, specific pedagogical practices that lead to creativity have not been identified (Sawyer, 2018). Everyone has capacity to be creative; although professional and extraordinary levels of creative achievement also require sufficient intelligence and domain knowledge (Sternberg and O’Hara, 1999; Jauk et al., 2013). How people actually develop creative expertise is still not well-understood, which may be due, in part, to issues with how creativity is defined and measured and the ways research areas are siloed.

Dietrich (2007), Glăveanu (2014b), and others have proclaimed that the field of creativity research is “in crisis” because of conceptual, methodological, and theoretical issues. First, the commonly accepted definition of creativity – as the ability to produce something novel (or original or unique) and useful (or valuable or meaningful) – does not sufficiently describe the concept’s multidimensionality (Dietrich, 2007; Fryer, 2012; Glăveanu, 2014b; Barbot et al., 2015). It limits attention to personal attributes and products produced, neglecting the physically and socially situated processes of problem finding and solving. Second, creative process research has shifted from a focus on identifying and modeling stages of creativity (for example, Wallas’s (1926) four-stage model) to mainly experimental studies of divergent thinking productivity (Dietrich, 2007; Glăveanu, 2014a; Barbot et al., 2015). The tendency to consider creativity an “intra-psychological activity” (Glăveanu, 2014b, p. 18) focuses on the outcome of creative thought as if cognition occurs in a vacuum devoid of bodily activity, material environment, and social-cultural context. Finally, creativity research is generally organized according the 4P’s: person; process; product; or press, social and/or physical contexts (Rhodes, 1961). Recently scholars have argued that this categorization is not sufficient to describe the field (Runco, 2007a; Lubart, 2017) and has enabled a disjointed understanding of creativity (Glăveanu, 2013). Although useful when creativity research was in its infancy, lack of significant progress in the creative process stream suggests 4P categorizations may be creating artificial barriers limiting scientific progress. New approaches are warranted that more deeply consider relationships between the 4Ps. EC and RECS perspectives may provide a framework that will move the field forward toward developing a new theoretical model better explaining the development of creative expertise and more useful for informing educational practices in formal and informal learning environments.

## A Conceptual Analysis of Radical Embodiment in Creativity

The concept of RECS is often described in terms of **4E** (***embodied, embedded, enactive, extended***) cognition, explaining ways the mind is not solely located in the brain but also involves the body and the body’s situation in the environment (Menary, 2010b; Newen et al., 2018). Although each of the E’s offers a different perspective on the nature of cognition, they are not entirely discrete; principles often overlap between them. ***Embodiment*** refers to how the body contributes to cognitive process and is based on the premise that the brain and body evolved together and are therefore intrinsically coupled. It considers the brain as part of a larger cognitive system, including the body’s nervous system and sensorimotor capabilities (Gallagher, 2015b), suggesting separation of body and brain is artificial. Gallagher (2015b) explains how body schema, a sensory-motor system that functions without explicit awareness (p. 24), structures our interactions in the world, shaping the mind at a fundamental level (p. 141). He suggests that once we understand how body schema shapes perception, we will uncover how it also shapes cognition as a whole, because perception is fundamental to cognition (p. 137). By this account, even abstract concepts are shaped by body schema. Lakoff and Johnson (1980, 1999) suggest we use linguistic metaphors to link abstract concepts with concrete, bodily experiences (such as “feeling warmth” to express affection). An embodied approach in creativity research might explore how, for example, gestures and postures influence perception during ideational processes. In his study of over 100 eminently creative people from diverse fields, Csikszentmihalyi (1996) found they often engage in common habits involving similar gestures or postures to support particular creative processes and “give their surroundings a personal pattern that echoes the rhythm of their thoughts and habits” (p. 127). Embodied creativity research might lend empirical insights into anecdotal creative habits.

***Embedded cognition*** describes coupling with environments (physical and socio-cultural), which shape, and are shaped by, the actors who inhabit them. This concept is inspired by Gibson’s (1977, 1979) ecological theory describing perception as direct (i.e., not requiring mental representation) and suggests actors perceive their environments in terms of affordances (opportunities for action) they provide to them. Affordances thus depend upon the unique bodily capabilities of the actor. Suchman (1987, 2007) and Hutchins (1995) extended these concepts to socio-cultural environments. Suchman (2007) studied human-machine interactions, realizing a photocopier help system’s communications breakdown was because its designer misunderstood the nature of action. She proposed a shift in the way plans are conceptualized, from “cognitive control structures that universally precede and determine actions to cultural resources produced and used within the course of certain forms of human activity” (p. 13). Planning, she explained, unfolds through a people’s interactions in their environments, as *discursive practice*. Hutchins studied a naval navigation team, concluding that cognition is a culturally constituted activity that depends upon the unfolding situation in which it occurs. He found that people off-load cognitive effort to their environments (through artifacts and other people) and establish dynamic social structures to improve cognitive load-balancing. More recently, Ingold’s (2002, 2010, 2013) study of artisans found their “craft” is not (solely) bodily skill, but emerges in a system of relationships and interactions situated in a particular socio-material environment. Embedded cognition approaches, such as Ingold’s, may yield new insights into the development of creative expertise acquired through situated practice.

***Enactive cognition*** argues that cognition is for action; through meaningful actions we make sense of our environment, allowing us to maintain our existence within it (Varela et al., 1974; Varela et al., 1991; Thompson and Stapleton, 2009). The enactive thesis, as developed by Varela et al. (1974, 1991), is influenced by *autopoiesis*, a concept that describes how organisms create their own experiences by initiating actions in environments. The enactive perspective considers people as “autonomous systems” who “regulate their interactions with the world in such a way that they transform the world into a place of salience, meaning, and value” (Thompson and Stapleton, 2009, p. 25). This idea aligns with how people often describe creative experience. For example, the architect Olga Aleksakova discusses thinking-in-action while cutting a foam block to create the design for a building (Yaneva, 2009). She explains how she began without a preconceived notion of the final form, rather it emerged though the intertwined processes of acting and perceiving. Each cut revealed to her new opportunities for subsequent actions to make the block more “beautiful.” Her creative process was an adaptive, reciprocal exchange between creator and creation. Examples like this one, suggest that creative expertise is developed through situated practice. Creative professionals develop a repertoire of actions as part of their practice, using them to initiate and sustain improvisational and adaptive interactions toward finding meaning in a creative situation.

Finally, ***extended cognition*** claims that thinking is distributed beyond the body (Clark and Chalmers, 1998, 2008). The things in our environment are incorporated into our cognitive systems, similar to the neuronal processes in the brain. This “first wave” extended cognition is committed to the *parity principle*, which argues that if non-biological agents extend cognition in a way that is functionally equivalent to intracranial processes, then that part of the world is part of the cognitive process (Clark and Chalmers, 1998, p. 8; Menary, 2010a) Clark (2008) uses an exchange between Richard Feynman and Charles Weiner to illustrate this concept. Feynman explains to Weiner that his notebook was not a *record* of his thinking, because his thinking happened outside of his head with pen and paper – the notebook *was part of* his thinking. “Second wave” extended cognition is focused on cognitive integration, explaining how bodily (internal) and non-bodily (external) aspects of cognition are integrated to form something new (Menary, 2010a). This wave does not rely on the parity principle, instead taking embodied-embedded cognition as a starting point. “Third wave” extended cognition proposes that cognitive integration is reciprocal, dynamic, and ongoing (Sutton, 2010). The boundaries of the mind are flexible and open-ended; cognitive processes unfold over time, involving the body, other people, and resources in the environment, shaped by socio-cultural practices. Significantly, the extended cognition approach argues that too much emphasis on neuronal and bodily aspects of cognition can obscure the importance of dynamic relationships between agent and artifacts. Of the 4Es, extended cognition is the most controversial, however it suggests that artifacts play a more significant role in creative cognition than typically considered in the literature. This has implications not only for how creativity is taught, but also for the design of learning spaces and other settings intended to support creativity.

Chemero (2009) frames RECS as a “variety of extended cognitive science” (pp. 31–32). He distinguishes RECS from Clark’s extended mind theory by its focus on a dynamical and non-linear coupled perspective, rejecting the “wide computationalism” and representational (linear) coupling that is typically the target of anti-extended-mind arguments. Baggs and Chemero (2018) also advocate for a “productive synthesis” between competing (and, he argues, complementary) ecological and enactive RECS approaches. The ecological perspective studies the environment in terms of opportunities (affordances) or constraints it offers for actors. The enactivist perspective, with its constructivist grounding, begins with the actor and is concerned by how it constitutes its own unique *umwelt^1^*. They suggest three definitions to clarify what is meant by “environment” in order to synthesize these perspectives. First the *physical world* describes the enduring structure of the environment irrespective of species inhabiting it. Second, *habit* describes the species-specific environment. Finally, *umwelt* describes the actor-specific environment. For the purpose of this conceptual analysis, the current state of embodied creativity research will be analyzed with respect to the 4Es from a synthetic RECS perspective as suggested by Chemero. Environment will be used as an umbrella term to describe both human-specific environment (habitat) and actor-specific environment (umwelt).

How people believe their bodies, artifacts, and environments shape creative processes is evident in personal accounts of creativity throughout history (Malinin, 2016), yet embodied creativity is a newly emerging research area, generally organized into two streams. The first stream involves experimental examination of embodied metaphors associated with creative thinking. These studies typically assess effects on ideational productivity of enacting metaphors through specific bodily movements. For example, free walking might enact the embodied metaphor *thinking outside of the box* (Leung et al., 2012; Kuo and Yeh, 2016). The second stream examines creativity from a dynamical systems perspective as an emergent phenomenon that is distributed between people and artifacts of the material environment. For example, musical creativity is examined as a system of emergent, dynamic interactions among musicians and instruments (Walton et al., 2014, 2015; Schiavio and Van der Schyff, 2018; van der Schyff et al., 2018) There are several key issues distinguishing these two approaches. First, the creative metaphor stream is concerned with understanding creative potential of the general population (typically college students). System approaches are often used to understand creative expertise by studying professionally or historically creative people. Second, the creative metaphor approach equates creativity with divergent or convergent thinking productivity, which are typically measured through Alternative Uses Test (AUT)^2^ or Remote Associates Test (RAT).^3^ The systems approach is concerned with the entire creative process, beginning with problem finding/framing through implementation. Where the embodied metaphor stream generally employs quantitative methods, the systems approach is more often qualitative or mixed methods — for example, integrating observation, sensors, and interviews to understand the dynamic interplay between people and artifacts with respect to creative processes. For the purpose of this conceptual analysis, creativity is defined as an iterative process that involves (a) identifying a problem or area of concern; (b) generating, testing, and elaborating on ideas toward developing a product (e.g., artifact, theory, methodology, system, performance, etc.); and (c) implementation and evaluation by others (for example, users and experts in the field). Divergent and convergent thinking are considered here as sub-processes, occurring throughout problem finding, generation/elaboration, and implementation.

### Embodied Metaphorical Creativity

Lakoff and Johnson (1980) proposed that mental concepts, such as metaphors, are directly tied to bodily experiences, suggesting a radical break with CC paradigms. Metaphors operate as mappings between source concepts, grounded in literal meanings (i.e., concrete, physical contexts), and abstract ideas. Embodied metaphors describe how literal and abstract meanings become intertwined, such that abstract concepts are metaphorically grounded in sensorimotor experiences. As embodied cognitive science has matured, some scholars argue that the embodied metaphor thesis is not radical, rather it is an example of grounded cognition that is compatible with CC models (Adams, 2010; Gallagher, 2015a; Shapiro, 2019). Nonetheless, a number of experimental studies examining embodiment in creativity have focused on how movement might enact embodied metaphors to enhance divergent and convergent thinking (Stanciu, 2015; Frith et al., 2019).

Many metaphors describe aspects of creative thinking, such as THINKING OUTSIDE OF THE BOX (Leung et al., 2012; Kuo and Yeh, 2016), BREAKING THE WALLS, THINKING FLUIDLY (Slepian and Ambady, 2012), MOVING FORWARD (Oppezzo and Schwartz, 2014), ON THE OTHER HAND (Leung et al., 2012), PUTTING TWO AND TWO TOGETHER (Leung et al., 2012), SQUEEZE YOUR HEAD (Kim, 2015), BEING OPEN (Hao et al., 2014, 2017; Andolfi et al., 2017), and BEING WARM OR COOL (Ijzerman et al., 2014). Two recent reviews found that, despite the small number of studies, embodied creativity metaphors did enhance divergent and convergent ideation, typically measured through AUT and RAT instruments (Stanciu, 2015; Frith et al., 2019). However, these experimental studies typically suffer from small sample sizes and lack of replication (see summery, Table 1). Studies examined how walking versus sitting (Leung et al., 2012; Oppezzo and Schwartz, 2014; Kuo and Yeh, 2016), arm movements/gestures (Leung et al., 2012; Slepian and Ambady, 2012; Kim, 2015; Wang et al., 2018), posture (Hao et al., 2014, 2017; Andolfi et al., 2017) and temperature (Ijzerman et al., 2014) might enact embodied metaphors immediately before or while engaging in creativity tasks.

**TABLE 1:** Embodied metaphorical creativity studies.

**Metaphor**	**Enacting bodily experience**	**Enhanced creative thinking**	**Sample**	**Authors**
Thinking outside the box	Free-walking	Divergent (originality)	102 college students	Leung et al., 2012
	Free-walking	Divergent (originality, fluency, flexibility)	64 college students; 32 adults aged 65 +	Kuo and Yeh, 2016
	Virtual free-walking	Divergent (originality)	73 university students	Leung et al., 2012
Moving forward	Treadmill walking	Divergent (originality. fluency); convergent analogy creation	48 college students	Oppezzo and Schwartz, 2014
Breaking the wall	“Breaking” arm movement	Divergent (originality, fluency, flexibility)	41 college students	Wang et al., 2018
On the other hand	Switching raised arms	Divergent (originality, fluency, flexibility)	40 college students	Leung et al., 2012
Putting 2 + 2 together	Moving arms together	Convergent thinking	64 college students	Leung et al., 2012
Thinking fluidly	Fluid arm movements	Divergent (fluency, originality, flexibility); convergent (remotely associations)	College students: 30 divergent; 150 converg.	Slepian and Ambady, 2012
Squeeze your head	Squeezing soft ball	Divergent thinking (originality, fluency, flexibility)	50 college students	Kim, 2015
	Squeezing hard ball	Convergent thinking	32 college students	Kim, 2015
Being open	Open body posture sitting	Divergent (originality, fluency, flexibility)	102 college students	Andolfi et al., 2017
	Open posture, positive emotion, standing	Divergent (originality) and associative flexibility	149 college students	Hao et al., 2017
	Seated with arm flexion, palm facing body	Divergent (originality and fluency)	100 college students	Hao et al., 2014
	Lying with arm extension, palm facing body	Divergent (originality and fluency)	100 college students	Hao et al., 2014
Being warm/cool	Warm environment	Divergent (fluency)	60 children aged 4–6	Ijzerman et al., 2014
	Cool thermal pad	Reacted faster to metaphorical statements, more abstract ideas	27 college students	Ijzerman et al., 2014
	Warm thermal pad	Higher quality, realistic ideas	33 college students	Ijzerman et al., 2014
	Holding warm cup	Relational thinking	23 college students	Ijzerman et al., 2014
	Holding cool cup	Divergent (originality)	33 college students	Ijzerman et al., 2014

Walking is a common habit people employ to help them come up with new insights when working on creative problems (Ghiselin, 1954; Buttimer, 1983; Solnit, 2001; Malinin, 2016). It has long held a special place in creativity; Aristotle’s Peripatetic School was so named because he walked with his students while philosophizing (Solnit, 2001). Walking has been used to enact metaphors for creative thinking, including THINKING OUTSIDE THE BOX, BREAKING THE WALLS, and MOVING FORWARD. Leung et al. (2012) found participants who engaged in creativity tasks while free-walking outside of a (5′ × 5′) box had better scores on both divergent (originality) and convergent thinking tasks than when sitting inside the box during tasks. Similarly, Kuo and Yeh (2016) found free-walking benefited ideational originality, fluency, and flexibility over rectangular (box-shaped) walking patterns for young and older adults. Virtual-reality (imagined) walking was also found beneficial. Participants whose avatars free-walked generated more original responses than those with avatars walking in rectangular patterns (Leung et al., 2012). In a study examining, BEAKING THE WALLS, participants used a game controller to move along a zigzagged corridor, turning their bodies to change direction (Wang et al., 2018). A wall appeared blocking the corridor in the “break” condition and participants destroyed it with the game controller in order to proceed. The “break” condition benefited ideational originality, fluency, flexibility, and persistence. Oppezzo and Schwartz (2014) hypothesized walking would benefit divergent thinking by helping participants MOVE FORWARD from one idea to the next. Walking on a treadmill facing a blank wall improved originality and fluency over sitting, but slightly reduced RAT scores of convergent thinking during and after walking. Free-walking outdoors and treadmill walking similarly benefited creative analogy generation, however walking outdoors contributed to more novel analogies, perhaps due to the additional sensory stimulation. In general, embodied metaphor studies demonstrate walking, particularly free-walking, improves divergent thinking but may slightly harm performance on memory intensive convergent thinking tasks.

Gestures, using arms and hands, were examined in a few studies of creativity metaphors. In an experiment examining ON THE OTHER HAND participants held one arm forward with palm up while completing creativity tasks (Leung et al., 2012). Switching hands improved ideational fluency, flexibility, and originality compared to putting the same hand forward in both trials. The metaphor PUTTING TWO AND TWO TOGETHER was enacted through an activity where participants moved stacks of coaster-halves located on the left and rights side of the table to the middle or simultaneously assembled the halves into a single stack (Leung et al., 2012). Combined stacking benefited convergent thinking whereas divergent thinking (fluency, flexibility, and originality) did not significantly different between conditions. Slepian and Ambady (2012) had participants trace two different drawings, one to elicit fluid arm movements (using curved lines) to enact THINKING FLUIDLY and one to create zigzagged movements (using straight lines and angles). Participants in the fluid condition showed improved fluency, originality, flexibility, and better connected remotely associated concepts. Analytical (convergent) thinking results did not differ between conditions. Kim (2015) examined a metaphor commonly used in Korea for fluid, divergent thinking, SQUEEZE YOUR HEAD. Squeezing a soft ball during TTCT and RAT assessments improved divergent thinking overall, and specifically fluency, flexibility, and originality. Squeezing a hard ball benefited convergent thinking. Interestingly, the participants for this study were British, where the metaphor is not commonly used. This might suggest a relationship between enacting a squeezing motor response and creative thinking. Or there may be another explanation. For example, Goldstein et al. (2010) had participants squeeze a ball with the left hand to artificially activate the right hemisphere prior to completing RAT, which improved performance.

Posture has been the focus of several studies examining, BEING OPEN. Andolfi et al. (2017) had participants sit in either an approach/expansive (with legs slightly spread and hands resting on legs) or avoidance/contractive (closed legs with arms crossed) posture while completing a series of divergent thinking tasks. Higher scores for fluency, flexibility, and originality were associated with the open posture. There was no significant difference for non-creative tasks or comfort between the two postures. Hao et al. (2017) had participants stand for their experiments examining relationships between posture, emotion, and creativity. Participants first watched videos to induce positive or negative emotions and then they completed AUT problems in either an expansive or contractive posture. In the open posture, participants stood with weight shifted to the front leg, arms down and hands slightly spread. In the closed position they stood with arms and legs crossed. Higher originality scores were associated when emotion and posture were compatible (positive-open or negative-closed). Participants also had greater associative flexibility in the positive-open condition and higher persistence in negative-closed conditions. In a prior study Hao et al. (2014) examined interactions between body and arm posture to enact approach or avoidance embodiment. They compared arm extension and arm flexion in seated and lying postures. They founded being seated with arm flexion was associated with higher AUT fluency and originality whereas when lying down the converse was true. In the seated condition, the arm was flexed with palm facing toward the participant. In the lying posture, the arm was flexed with palm upward, facing away from the participant, but in the arm extended position the palm was downward, facing toward the participant. Proximity between palm and body seemed to enact an approach motor action, found in prior research to improve global processing, abstract thinking, and making connections between remote concepts (Andolfi et al., 2017).

Environmental conditions may also enact embodied metaphors (Jia et al., 2009; Ijzerman et al., 2014). People commonly believe certain places help them be more creative (Kristensen, 2004; Vohs et al., 2013; Malinin, 2016) and there is some evidence that atmospheric conditions alone can elicit embodied metaphors benefiting creativity. Ijzerman et al. (2014) explored how temperature might elicit BEING WARM (relational thinking) or BEING COOL (conceptual distance) in a series of three experiments. The first study used environmental temperature, conducting studies with children in rooms that were cool (15–19°C/59–66° F) and warm (21–26°C/70–80° F). Children created more fluent drawings in the warm room. In their second study, they used thermal pads to heat or cool adult participants for 3 min prior to completing creative tasks. Participants in the cool condition reacted faster to metaphorical statements during a computer task measuring how fast they switch their interpretive frame. There was no reaction time difference for factual statements between conditions. Therapeutic pads were also used during tasks where participants came up with gift ideas for friends and strangers. In the warm condition, participants developed higher quality, realistic gift ideas for both friends and strangers. In the cool condition, gift ideas were more abstract. In another experiment, the researchers had participants hold a cup filled with a warm or cool beverage while completing a category inclusion task and while coming up with creative names for a new pasta. When holding the warm cup, participants demonstrated more inclusive categorization whereas holding the cool cop benefited originality. These series of experiments suggest environmental warmth may benefit fluidity and relational thinking during creativity whereas cooler temperatures may be useful to elicit more abstract, original thinking.

There does seem to be a small but compelling body of research suggesting the role of the body in enacting metaphors for creative thinking, however more research is needed that replicate results. Nonetheless, these findings align with “traditional” psychology studies demonstrating, for example, benefits of physical activity, including walking (Colzato et al., 2013; Oppezzo and Schwartz, 2014), how movements can activate the right hemisphere to enhance divergent thinking (Goldstein et al., 2010; Mihov et al., 2010), and how the ambient conditions in a setting can benefit creativity through processing disfluency (low level distraction) (Mehta et al., 2012). The embodied metaphor stream underscores how the body constrains cognition, however this is not necessarily incompatible with representational models of cognition. RECS suggests that through movement, such as gesture, the body is able to express abstract concepts without the need for mediating verbal representation (Slepian and Ambady, 2012). The embodied metaphor studies, however, generally focused on ways the body might bias abstract thinking or *mindset* (disposition/intent toward a creative situation) to improve divergent or convergent thinking. This aligns with what Gallagher (2015b) refers to as *minimal embodiment*. Yet there is still potential for advances in this research stream to inform learning space designs and pedagogical practices that consider impacts of environmental qualities, movement, posture, and gesture on different creative processes, including divergent and convergent thinking and conceptual combination (analogical/metaphorical thinking).

Metaphorical thinking is understood to be central to creativity (Kazmierczak, 2003), yet embodied metaphor studies have not attempted to enact metaphors to influence creative insight on ill-defined problems. This is an important area that might inform pedagogical practices to enhance creativity. For example, there are numerous anecdotes suggesting conceptual combination leading to insight is often immediately preceded by embodied, sensorimotor experience. Philo Farnsworth had the idea to project moving images line-by-line while he was plowing a field, leading to the invention of television (Thomas, 2004). George de Mestral was picking burrs off of his dog when came up with the inspiration for Velcro (Hargroves and Smith, 2006). There is an untapped potential for experimental studies of embodied metaphor to examine how sensorimotor experiences might *constitute* new knowledge leading to creative breakthough. The experimental work of Slepian and Ambady (2014) creating novel embodied metaphors could provide a direction for future research –and would align more closely with the RECS approach. The pair taught participants novel metaphors pertaining to weight and time and found these influenced estimates of physical weight (when holding a book), suggesting embodied simulation. Metaphors did not influence weight perceptions when looking at photos of books, suggesting results were not because of semantic association. Studies examining novel metaphor creation during authentic problem solving might provide greater insight into the role of sensorimotor experience in enacting new insights. This could help to answer the question of whether creativity requires internal representations or if creativity is distributed beyond the brain, to include the body and environment.

### Creativity-in-the-Wild

A core argument of RECS is that human cognition is situated activity, characterized as a dynamical system encompassing brain, body, and world. Dynamical systems approaches employ “mathematical abstraction that unambiguously describes how the state of some system evolves over time” (Beer, 2014, p. 134). It includes states, time, and operators that transform a state at one time to another state at a different time. This is an alternative to experimental embodied-metaphor approaches that focus attention to the role of action in ideation processes, but do little to dispel the notion that creativity happens *solely in the head*. Since the 1970s, cognitivist approaches, which imply creativity starts with a mental idea subsequently imposed upon a material world, have supplanted empirical study of physically-situated, dynamic practices, such as tool use during creativity (Baber, 2019). A notable exception, Schön (1983) observed architects and others in their workplaces, finding that people *think-in-action*, by “conversing” with the materials of a creative situation (p. 175). Csikszentmihalyi (1990) interviewed over 100 creative professionals who described a similar process, which he called *flow*. Tools, materials, and settings are critical for initiating and sustaining a flow state – which is when people feel most creative (for example, pp. 54–58 and p. 120). Woodman and Schoenfeldt (1990, p. 10) argued decades ago that to understand creative behavior, one must begin by understanding the “organism-in-its-environment.” There are a few recent efforts incorporating dynamic systems theory (DST) in studies of performing arts and design, yet domain general studies remain rare – despite evidence that creativity unfolds through person-environment interactions with tools, materials, and even workplace settings during creative processes (Sennett, 2008; Pallasmaa, 2010; Ingold, 2013; Malafouris, 2013; Glăveanu, 2014a).

Complex-systems theories of creativity emerged in the 1980s, acknowledging creativity as a socially situated process. Psychological studies recognized that people work within particular social structures, which influences creativity (Amabile, 1983, 1996) and creative products are evaluated by members of a person’s field through consensual assessment (Feldman et al., 1994). The material environment remains mostly disregarded. Amabile (1998) argued the physical workplace setting does not play any significant role in creativity. Csikszentmihalyi (1996, p. 135) suggested the material environment is important for creativity, but it may be impossible to empirically explain how it might catalyze creative processes. Developments in 4E cognition have informed complex systems frameworks for two recent studies of embodied creativity. Both examined the materiality of creative practices compared across domains of visual and performing arts, design, writing, and science. First, Glăveanu et al. (2013) proposed an action framework based on Dewey’s (1934) experiential learning theory to guide qualitative analysis of interviews conducted with 60 artists, musicians, designers, writers, and scientists. They propose that creative acts typically begin with an impulse, which varies by domain (e.g., to express, to solve a problem, etc.). Material and methodological constraints on creative acts constitute experiences through which the creator gains awareness until eventually achieving emotional fulfillment and professional satisfaction. Malinin (2016) included the architectural environment in her analysis of accounts by creative professionals for evidence of embedded, embodied, and enactive cognition. The resulting theoretical framework, grounded in Gibson’s (1977) affordance theory, describes how creative niche construction (*umwelt*) is constituted through person-environment coupling. She found creatives habitually exploit features and qualities of their material environments to engender, sustain, and curtail different modes of creativity, enhancing creative productivity. The materiality of creative practices across domains is apparent in both studies of creativity *in situ*, suggesting it may be time to finally leave behind purely mental models that focus on divergent and convergent thinking in favor of DST approaches. Furthermore, the theoretical frameworks developed from these studies suggest that person-material-environment interactions – doing-and-undergoing (Glăveanu et al., 2013) or perceiving-in-action (Malinin, 2016) – constitute transformative creative experiences.

The idea that creativity is *emergent* and *distributed* between people and artifacts is not a new concept in improvisational performing arts, such as comedy theater (Sawyer, 1999; Sawyer and DeZutter, 2009), music (Walton et al., 2014, 2015, 2018; Schiavio and Van der Schyff, 2018; van der Schyff et al., 2018), or partner-dance (Kimmel et al., 2018; McClure, 2018). Sawyer’s (1999) seminal study of theatrical improvisational was informed by Hutchins’s (1995) theory of distributed cognition. Hutchins argued that the best way to understand embodied activity is to study real world processes by observing people in their workplaces. Through this methodology he illustrated how real-world problem solving, which he called “cognition-in-the-wild,” is distributed between people and artifacts. Improvisational creativity is unique; the process *is* the creative product and activities are interactional and unpredictable (Sawyer and DeZutter, 2009). Temporal, observational studies of improvisational performances reveal some common principles about person-environment interactions during this type of creativity:

• Creative synergies emerge without prior planning or scripting and cannot be attributed to the intentions or actions of any particular participant (Sawyer and DeZutter, 2009; Kimmel et al., 2018).
• There is moment-to-moment contingency where each action depends on the one just prior and any action can be changed by subsequent actions (Sawyer and DeZutter, 2009; van der Schyff et al., 2018).
• Cultivation of embodied perception — acquired through a repertoire of bits/motifs (relatively stable interactional routines) — increases potential for novelty (Sawyer and DeZutter, 2009; Kimmel et al., 2018; McClure, 2018).
• Interactions constitute micro-affordances, which enact meaning for participants (Sawyer and DeZutter, 2009; Kimmel et al., 2018).
• Creativity is a complex system constrained by interactions (Sawyer and DeZutter, 2009); constraint lies in the structure of the partnership (Walton et al., 2015; McClure, 2018; van der Schyff et al., 2018) and the setting (van der Schyff et al., 2018; Walton et al., 2018).
• Higher level structures emerge, exhibiting global system behavior (Sawyer and DeZutter, 2009); the creative group becomes so tightly coordinated that they behave as a single entity and not a collection of individuals (Walton et al., 2014, 2015).
• Social and material interactions are reciprocal; the creative process is transformative (Glăveanu et al., 2013; van der Schyff et al., 2018).

Design creativity has also been examined as a type of improvisational performance between actor and materials of a creative situation (Schön, 1983; Pereira and Tschimmel, 2012; Choi and DiPaola, 2013; Rietveld and Brouwers, 2017; Baber et al., 2019). Baber et al. (2019) used a RECS framework to study jewelry design. They analyzed data from interviews, motion capture, and sensors fitted to tools to understand how artifacts (tools, equipment, materials, and workplace) shape creative activity, finding jewelry design involves more technological reasoning than abstract reasoning. They propose that (a) creativity is a physical act where action and perception are intertwined, (b) creativity emerges through incremental insight as responses to changing situational cues, (c) it involves a repertoire of responses to situational cues and constraints, and (d) constraints are necessary, but too many inhibit creativity. Like research on improvisational arts, their study demonstrates how creativity is emergent and distributed between actor and artifacts. Pereira and Tschimmel (2012) also found jewelry making to be an emergent phenomenon. They suggest perception is at the core of creativity, which might emerge through *confused perception* (such as Beethoven’s deafness), *malfunctioning perception* (such as with mental illness), and *intentional perception*, developed through expertise and use of strategies like associative and analogical thinking. Rietveld and Brouwers (2017), in their ethnography of architectural practice, noted that architects also continually shift perspectives to perceive new affordances in a creative situation. Some ways this is done in both jewelry making and architecture include ideation drawing and tool use.

Baber et al. (2019) propose that designers *instantiate events* by creating ideation sketches. Chemero (2000), Chemero et al. (2003) defines events as “changes in the layout of affordances” in the actor-environment system. Affordances are relations between the particular skills and abilities of the actor with respect to features and qualities of the environment (Chemero, 2003). Ideation drawings create changes in the affordances of the design situation, helping the designer explore concepts and perceive new opportunities to act upon. Drawings can expand the problem space because they allow designers to abstract ideas, emphasizing or disregarding aspects of the problem (Pereira and Tschimmel, 2012). In architecture, drawings and model making are both abstractions; the designer is unable to perceive affordances from the creative product itself because others build it after the design is finalized. Construction drawings are a blueprint from which to build the final product, but ideation drawings are physical forms of problem solving. Models serve a similar role in architecture, as abstracted, physical ideation (Yaneva, 2009, p. 57; Malinin, 2016). In jewelry making, however, the designer is able to also directly work with the materials of the final product.

Research on improvisational performance and design practices provides evidence that creativity does not begin with an idea in the head that is subsequently realized; it emerges through interactions with others and artifacts of the material environment. There is some evidence to suggest that other forms of creativity are similarly emergent and distributed, such as scientific (Watson, 1968; Gruber, 1981; Glăveanu et al., 2013; Malinin, 2016) and literary (Kipling, 1937; Glăveanu et al., 2013; Malinin, 2016) domains. Studies of real-world creativity also demonstrate how the phenomenon is transformative. Expertise changes perceptional abilities, instantiating events change affordances to be perceived, affordances provide opportunities for actions that shape creative products, which, in turn, change the creator. Creativity, thusly, can be characterized as a dynamical system encompassing brain, body and world — in line with the RECS perspective. However, the question of whether creativity requires mental representation remains.

In *The Reflective Practitioner* (which argued against Technical Rationality, rooted in CC)^4^ Schön (1983) stated that designers “know more than they can say” (p. viii), that their knowing is embedded in their practice of creating (p. 60). Baber (2015, 2019) makes a similar argument, using examples from jewelry design to suggest how creativity need not rely on stored mental representations (Baber, 2015, pp. 33–34). He proposes creativity emerges through “opportunities within a space of constraints” (Baber, 2019, p. 229). By manipulating tools and materials, through “routine, improvised, or opportunistic action,” people are changing the information they embody (p. 233). He illustrates how people (a) arrange their workspace to support creative activities, (b) arrange components of the design project, (c) arrange the body in anticipation of completing a task, (d) and respond to perceived changes in the materials or tools of a creative situation to suggest that creativity is a form of *technological reasoning* (p. 232). Similarly, Rietveld and Brouwers (2017) use their study of architectural creativity to argue that “the dichotomy between ‘lower’ and ‘higher’ cognition is largely artificial…” (p. 545). They advocate for a framework focused on skilled intentionality that integrates research on material affordances (the focus of most design creativity research) and social coordination (the focus of most improvisational creativity research). They suggest creativity can be characterized by skilled activities unfolding in a complex situation, rich with affordances. Both proposals suggest creativity might be best understood as a self-organizing dynamical system. Baber focuses discussion around how the designer tends to navigate multiple affordances to “minimize risk, effort and other costs and maximize benefit and quality of the outcome” (Baber et al., 2019, p. 283) Whereas Rietveld and Brouwers use the concept of *optimal grip* to describe a type of skillful coping in the presence of multiple affordances, as an optimal actor-environment relationship. They explain how architects tend toward optimal grip in visual perception, optimal grip on the design project, and optimal grip on “how to design.” Together these perspectives suggest how creativity is a form of distributed cognition involving bodily, material/technological, socio-cultural, and temporal dimensions, presenting a similar thesis, focused around the role of perception-in-action, to account for creativity in absence of stored mental representation.

Dynamic systems theory approaches to creativity, particularly music and design domains, have yielded new insights into the role of the socio-material environment in the development of creative expertise. Such insights are critical for better understanding how to teach skills associated with creativity. Schön’s (1983) seminal work describing thinking-in-action, although not specifically informed by DST, remains influential for informing pedagogical practices in design, nursing, and teacher education (Burton, 2000; Webster, 2008; Sator and Bullock, 2017). More research is needed, informed by 4E cognition and DST, to identify specific pedagogical practices that lead to creativity. A recent article by Schiavio and Van der Schyff (2018) outlines a 4E framework to inform music pedagogy that bridges concepts of autopoiesis and DST to describe the reciprocal nature of social and material interactions involved in musical skills development. Theirs is an important first step that can help to inform future efforts in other domains, as well as across domains more generally.

## Discussion (Embodied Creativity Matters)

Creative thinking is an essential skill for the future workforce (Barbot et al., 2015; Jules and Sundberg, 2018) and crucial for solving wicked^5^ societal problems, such as overpopulation, poverty, and climate change. However, creative process research has largely stalled (Dietrich, 2007; Fryer, 2012; Glăveanu, 2014b; Barbot et al., 2015) and has not been particularly useful for informing educational psychology or pedagogical practices (Plucker et al., 2004; Glăveanu, 2014b; Sawyer, 2018). The field of creativity research is in crisis because, Glăveanu (2014a, p. 2) argues, “one of the most pervasive separations creativity researchers make is between creator (or his brain) and the social and material world.” The body plays a key role in cognition (Gallagher, 2018), which has implications for embodied approaches to learning and development of professional expertise, including creative talent, and the design of environments where these occur. Thus there remains an untapped potential for embodied creativity to inform the design of learning spaces and instructional practices, better preparing the future workforce to solve critical societal and ecological *grand challenges*.

Analysis of the current state of embodied creativity research suggests some promising directions —but also that there is much work to be done to define and focus efforts in this nascent sub-field of creativity. Metaphorical creativity approaches mainly align with a *weak embodiment* perspective and grounded cognition. They do provide compelling evidence that the body shapes *creative mindset*, or disposition toward a creative situation. To date, this research stream is most relevant for informing learning environment and workplace strategies to improve *creative potential*. For example, building designs might take into account how thermal control could be strategically used to enhance creative thinking. Room configurations and building landscapes might be designed to encourage free walking. Furnishings could facilitate different postures, gestures, or walking movements (such as treadmill desks) to support divergent, convergent, or associative thinking processes. Such efforts, however, would need to include educating users about how to exploit these features of their workplace or instructional settings to improve creative productivity. Some of the embodied metaphors studied align with common habits that historically creative people incorporate into daily practice (such as walking or holding a warm cup), suggesting future research efforts have the potential to inform specific instructional practices, such as how to reduce creative fixation. Finally, metaphorical creativity research could integrate a strong embodiment (RECS) perspective to examine “mental” processes commonly associated with creativity, such as imagination, incubation, and conceptual combination. Slepian and Ambady (2014) demonstrated how creation of novel embodied metaphors provides evidence of embodied simulation. Approaches such as theirs could lend new insights into, for example, the little understood phenomenon of creative incubation leading to insight.

Recent dynamical systems approaches in creativity research, primarily in improvisational arts and design domains, more closely align with RECS. This stream of research also has the greatest potential to address the conceptual, methodological, and theoretical issues creating the present day “crisis” in the field. First, research in this area reveals that the common definition of creativity, as the ability to produce something novel and useful, does not adequately describe creativity-in-the-wild (i.e., real-world creativity). Creativity is situated practice; it involves embodied experiences and is embedded in socio-material environments. Dynamical systems approaches also describe how creativity is enacted through person-environment interactions and distributed among technological-material artifacts and other people. It could also address the emergent nature of creativity, constituted through reciprocal and transformative social and material interactions. Such approaches have strong potential to yield new insights relevant for informing pedagogical practices to teach creativity. In acknowledging 4E creativity, it follows that traditional methodologies, including the dominant focus on

experimental approaches using AUT and RAT assessments, are simply not sufficient (and probably not even appropriate) to fully understand creative skill development. Finally, theoretical approaches — such as Baber’s (2019) concept of creativity as *technological reasoning* or Rietveld and Brouwers’s (2017) notion that it involves *skilled intentionality toward optimal grip* — begin to address the demand for a new theoretical framework that integrates the historically siloed 4Ps of creativity research.

What, then, are next steps for embodied creativity research? First and foremost, a new definition of creativity is needed to describe creativity as situated practice, emerging through person-environment interactions (material/technological as well as socio-cultural). Second, new conceptual and methodological tools must be adopted, including developmental and dynamical systems approaches, to better understand person-environment relationships (for example, how and when technological artifacts are instrumental) throughout the creative process — as well as how these relationships evolve with acquired domain expertise. Third, a common theoretical framework, informed by RECS, would provide a starting point to integrate research in the 4P’s, including the often overlooked workplace or classroom physical environment (creative press) and the emerging neuroscience of creativity research stream (creative process). Dynamical systems approaches highlight the role of perception in creativity. Skilled perceptual abilities can be taught, as is a focus of pedagogical techniques in design programs. Yet, there is little understanding of how perceptual abilities lead to creative expertise. Architectural settings, such as learning spaces, shape sensorimotor experiences, which, in term, shape perceptions of affordances available to inhabitants. However, creative press research has prioritized socio-cultural over material-technological environments. Neuroscientific approaches propose a predictive processing (PP) model^6^ of creative perception (Gabora, 2011; Dietrich and Haider, 2015, 2017). Although PP is typically considered in representational terms (Kirchhoff, 2017), and, at best, a weak form of embodied cognition (Gallagher, 2015b). Synergies between PP and studies of creativity as a self-organizing system of mind, body, and socio-physical environment (Walton et al., 2014, 2015; Baber, 2019; Baber et al., 2019) might present a starting point. Developing creativity in educational (or workplace) settings is a complex endeavor — and one that has not benefitted enough from creativity research (Barbot et al., 2015; Sawyer, 2018). For creativity to become a learning competency, essential for the future workforce, research approaches must consider its multidimensionality to effectively inform pedagogical practices — and acknowledge the role of the body, artifacts, and the larger socio-material environment in the development of creative expertise.

## Author Contributions

The author confirms being the sole contributor of this work and has approved it for publication.

## Conflict of Interest

The author declares that the research was conducted in the absence of any commercial or financial relationships that could be construed as a potential conflict of interest.

## Footnotes

1 von Uexküll (1926/2010) described *umwel*t as the “surrounding environment” of an organism that is structured through its senses and abilities. Every organism has its own umwelt, even if they occupy the same space. The living organism is always at the center of its umwelt and is conceptually bound to it.

2 AUT measures divergent thinking (fluency, originality, flexibility, and elaboration of ideas) by coming up with alternative uses for common household objects, such as a brick.

3 RAT measures convergent thinking by identifying a single word that links together a list of three common stimulus words that are seemingly unrelated. For example, “box” links the stimulus words “square,” “cardboard,” and “open.”

4 See Chapter Two: From Technical Rationality to Reflection-in-Action.

5 Rittel and Webber (1973, 1984) define wicked problems as complex societal challenges that are difficult to solve because knowledge is incomplete or contradictory, the problems are connected to other problems (and solving them can create new problems), there many people and other factors involved, and there is an economic burden in solving them.

6 Predictive processing models suggest that the purpose of the brain is to make predictions of the world, and that these predictions inform and constrain perception (Clark, 2015; Dietrich and Haider, 2017).
